# Dietary supplementation of cinnamon and turmeric powder enhances growth performance, nutrient digestibility, immune response, and renal function in broiler chickens

**DOI:** 10.1016/j.psj.2025.105556

**Published:** 2025-07-11

**Authors:** Khanzada Khan, Hanan Al-Khalaifah, Nazir Ahmad, Muhammad Tahir Khan, Rasha Alonaizan, Rifat Ullah Khan, Shabana Naz, Ala Abudabos, Ibrahim A. Alhidary

**Affiliations:** a Department of Animal Nutrition, Faculty of Animal Husbandry & Veterinary Science, The University of Agriculture, Peshawar, Pakistan; b Environment and Life Sciences Research Center, Kuwait Institute for Scientific Research, Safat, Kuwait; c Department of Zoology, College of Sciences, King Saud University, Riyadh, Saudi Arabia; d Physiology Lab, College of Veterinary Sciences, Department of Animal Husbandry and Veterinary Sciences, The University of Agriculture, Peshawar, Pakistan; e Department of Zoology, Government College University, Faisalabad, Pakistan; f Department of Agriculture, School of Agriculture and Applied Sciences, Alcorn State University, 1000 ASU Drive, Lorman, Mississippi 39096-7500, USA; g Department of Animal Production, College of Food and Agriculture Sciences, King Saud University, Riadh, Saudi Arabia

**Keywords:** Broiler chickens, Cinnamon powder, Turmeric powder, Growth performance, Immune response

## Abstract

This study investigated the effects of dietary cinnamon and turmeric powder supplementation on growth performance, carcass traits, nutrient digestibility, immune response, and renal function in broiler chickens. A total of 300 ROSS 308 broiler chicks were randomly allocated to five dietary treatments: control, 0.5 % cinnamon, 1.0 % cinnamon, 0.5 % turmeric, and 1.0 % turmeric powder, each with six replicates of 10 birds. Birds were reared from day 1 to 42 under standard management, with treatment diets offered from day 6 onwards. Results revealed that birds receiving 1.0 % cinnamon and 1.0 % turmeric exhibited significantly higher body weight gain, feed intake, and improved feed conversion ratio throughout the trial (*P* < 0.01). These groups also showed superior carcass traits, including higher dressing percentage and breast/thigh weights, along with reduced abdominal fat. Apparent digestibility of dry matter, crude protein, fat, and fiber was markedly enhanced in the high-dose groups. Immune response, as assessed by antibody titers against infectious bursal disease (IBD), infectious bronchitis (IB), Newcastle disease (ND), and sheep red blood cells (SRBC), was significantly elevated in the 1.0 % cinnamon and turmeric groups. Additionally, these treatments resulted in significantly lower serum uric acid levels, indicating improved renal function. Overall, the inclusion of 1.0 % cinnamon or turmeric powder improved growth, health, and nutrient utilization in broilers.

## Introduction

The global poultry industry is continually exploring sustainable alternatives to antibiotic growth promoters (AGPs) in response to increasing regulatory restrictions and consumer demand for antibiotic-free meat (Rashid et al., 2023; Ahsan et al., 2024; Dablool et al., 2024; Mahmood et al., 2024; Ali et al., 2025; Satti et al., 2025). Phytogenic feed additives (PFAs), derived from herbs, spices, and essential oils, have gained considerable attention due to their ability to improve growth performance, nutrient digestibility, immune status, and overall health in broiler chickens (Ajmal et al. 2023; Aljohani 2024; Basit et al., 2025; Rasheed et al., 2024; Uzair et al., 2025). Among these, turmeric (*Curcuma longa*) and cinnamon (Cinnamomum spp.) are two promising phytobiotics, known for their bioactive compounds with potent antioxidant, anti-inflammatory, antimicrobial, and immunomodulatory properties (Raza et al., 2016; Hafeez et al., 2020; Ullah et al., 2022; Khan et al., 2022).

Turmeric contains curcuminoids, especially curcumin, which exhibits a broad spectrum of biological activities. In poultry nutrition, turmeric has been reported to improve feed conversion ratio (FCR), enhance intestinal morphology, and boost immunity (El-Gogary et al., 2025). Curcumin enhances digestive enzyme secretion and reduces lipid peroxidation in tissues, thereby promoting better nutrient utilization and oxidative stability of meat (Khan et al., 2012). Cinnamon, on the other hand, is rich in cinnamaldehyde and eugenol, compounds known to enhance digestion and exert antibacterial effects against intestinal pathogens such as *Escherichia coli* and *Clostridium perfringens* (El-Hamid et al., 2024). Its addition to broiler diets promotes better growth, digestive health, and immunity (Saied et al., 2022). These improvements are attributed to enhanced digestion, improved gut microbiota balance, and increased nutrient absorption.

The antioxidant properties of turmeric and cinnamon may offer protective effects by scavenging free radicals and reducing oxidative damage. Curcumin has been shown to downregulate pro-inflammatory cytokines and modulate the activity of enzymes involved in oxidative stress, thereby supporting kidney function (Khan et al., 2012). Cinnamon also exhibits nephroprotective effects through its antioxidant and anti-inflammatory activities (Alshahrani et al., 2021). However, the response may be dose-dependent, warranting the investigation of different inclusion levels. Thus, integrating these additives into broiler diets may enhance both humoral and cellular immunity.

Despite the promising individual effects of turmeric and cinnamon, limited research has evaluated their comparative or synergistic effects at different dietary levels (Naderi et al., 2014). Furthermore, comprehensive studies that simultaneously assess their impact on growth performance, carcass traits, nutrient digestibility, renal function, and immune responses are scarce. Given their diverse bioactive profiles, it is plausible that optimized levels of these spices could confer multiple benefits in broiler production. Therefore, the present study aims to evaluate the effects of different levels of dietary turmeric and cinnamon powder supplementation on growth performance, carcass characteristics, nutrient digestibility, renal function, and immune responses in broiler chickens.

## Materials and methods

### Bird management

The research trial was conducted in an open-sided poultry house equipped with a cross-ventilation system. A total of 300 day-old broiler chicks (ROSS 308), uniform in initial body weight, were procured from a commercial hatchery. Upon arrival, the chicks were reared on a commercial starter diet for the first five days. During the brooding period, artificial heating was provided using electric brooders to maintain the recommended brooding temperature of 33.0 °C at day one. The temperature was then gradually reduced by 5 °C each week until it stabilized at 23.0 °C by the end of the third week.

The chicks were randomly allocated into five dietary treatment groups. Each treatment was replicated six times, with 10 chicks per replicate, following a Completely Randomized Design (CRD). Birds were reared in floor pens (4 × 4 feet each), which were bedded with a uniform layer of clean, dry sawdust litter to a depth of approximately 3–4 inches to provide comfort and absorb moisture. Continuous bright lighting was maintained at a 23-hour light and 1-hour dark cycle (23L:1D) throughout the experimental period.

### Experimental diets

The study included five dietary treatment groups: a control group (C) that received only the basal diet; T1, which received 0.5 % cinnamon powder (CNP); T2, with 1.0 % CNP; T3, supplemented with 0.5 % turmeric powder (TP); and T4, with 1.0 % TP. These diets were fed from day 6 to day 42 of the experiment. Feed and clean drinking water were provided *ad libitum* throughout the trial period. Some of the important bioactive compounds in turmeric and cinnamon are given in Table 2. To ensure uniform distribution of the phytogenic powders (CNP and TP), the required quantities were first premixed with a small portion of the basal diet to form a concentrated blend. This premix was then thoroughly mixed with the remaining feed using a horizontal feed mixer for 15 minutes to achieve homogeneity. The mixing procedure followed standard feed manufacturing practices, and all diets were prepared fresh every two weeks to maintain consistency and minimize nutrient degradation.

Dried cinnamon bark and turmeric rhizomes were purchased from a local market, and their botanical species were authenticated by a qualified botanist. The plant materials were dried in a hot air oven at 105 °C for 3 hours and then ground separately using an electric grinder to obtain fine powders. These were stored in airtight containers at room temperature until use. Prior to inclusion in the diets, the proximate composition of both powders was determined.

The proximate chemical composition of cinnamon powder (CNP) was as follows: crude fiber 14.6 %, ash (mineral matter) 11.3 %, crude protein 6.6 %, ether extract 7.5 %, moisture 9.5 %, and carbohydrates 50.5 %. For turmeric powder (TP), the composition was: crude fiber 9.5 %, ash 8.7 %, crude protein 7.9 %, ether extract 8.1 %, moisture 10.6 %, and carbohydrates 55.2 %.

All experimental diets were formulated using corn and soybean meal as the primary ingredients to meet or exceed the nutritional requirements recommended by the National Research Council (NRC, 1994). Two distinct diets were formulated corresponding to the broiler growth phases: a starter diet (day 1–21) and a grower diet (day 22–42). The ingredient composition and calculated nutrient content of the experimental diets are provided in Table 1.

**Table 1:** Basal diets.

Growth phase	Starter (0-21 day)	Grower (22-42 day)	
Ingredients	Inclusion level %	Inclusion level %	
Ground Yellow corn grains	58.07	62.07	
Soybean meal (45 % CP)	34.0	31.0	
Fish meal (60 % CP)	2.00	—	
Vegetable oil	2.00	3.00	
Sodium Chloride	0.30	0.30	
Dicalcium Phosphate	1.70	1.70	
Ground Limestone	1.00	1.00	
Vitamins+Minerals Premix	0.50	0.50	
DL-Methionine	0.21	0.21	
L-Lysine	0.12	0.12	
Coccidiostat	0.10	0.10	
Total	100	100	
Calculated analysis			
Nutrient / constituent	Percent availability	Percent availability	
Dry Matter	89.63	89.76	
Crude Fibre	3.01	3.01	
Crude fat	11.14	11.62	
Crude Protein	21.78	19.74	
Metabolizable Eenergy Kcal/kg	2968	3062	
Calcium	1.02	0.930	
Available phosphorus	0.479	0.417	
L-Lysine	1.36	1.19	
DL-Methionine	0.566	0.517	
Minerals per kg			
Iron	60mg	Thiamine	3mg
Manganese	80mg	Riboflavin	6mg
Zinc	60mg	Pyridoxine	5mg
Cobalt	0.2mg	Cyanocobalamin	0.03mg
Cupper	5mg	Pantothenic acid	25mg
Iodine	1mg	d-biotin	0.05mg
Choline chloride	200mg	Folic acid	1mg
Selenium	0.15mg	Cholecalciferol	2400IU
Retinol	12000IU	Menadione	4mg
DL- α-tocopherol	50mg

**Table 2:** Bioactive compounds in turmeric and cinnamon.

	Bioactive Compound	Estimated Content	Determination Method
Turmeric	Curcumin	3.1, % of dry weight	HPLC (UV detection at 425 nm)
	Total curcuminoids	4.5, % of dry weight	HPLC
	Total phenolics	56.7, mg GAE/g extract	Folin–Ciocalteu assay
	Total flavonoids	35.6, mg QE/g extract	AlCl₃ Colorimetric assay
	Antioxidant activity (DPPH)	65.7, % inhibition	DPPH assay (abs at 517 nm)
	Volatile oils (e.g., turmerone)	5.6, % of dry weight	Steam distillation + GC-MS
	FRAP value	234, µmol Fe²⁺/g extract	FRAP assay
Cinnamon			
	Cinnamaldehyde	65, % of essential oil	GC-MS
	Eugenol (*C. zeylanicum*)	7.5, % of essential oil	GC-MS
	Total phenolics	85, mg GAE/g extract	Folin–Ciocalteu assay
	Total flavonoids	56, mg QE/g extract	AlCl₃ Colorimetric assay
	Coumarin (*C. cassia*)	3.4, mg/g	HPLC or GC-MS
	Antioxidant activity (DPPH)	67, % inhibition	DPPH radical scavenging assay
	FRAP value	550, µmol Fe²⁺/g extract	FRAP assay

### Growth performance parameters

Body weight and feed intake were recorded weekly to calculate weight gain and feed conversion ratio (FCR). Body weight gain (BWG) was calculated by subtracting the initial weight from the final weight of birds in each replicate. Feed intake (FI) was determined by subtracting the residual feed from the total feed offered. FCR was calculated as the ratio of feed intake to body weight gain (g/g).

### Carcass traits evaluation

At the end of the 42-day experimental period, one bird closest to the average body weight was randomly selected from each replicate (n = 4 per treatment) for carcass trait evaluation. Prior to slaughter, birds were fasted for 6 hours with access to water. The selected broilers were humanely slaughtered following standard halal slaughtering procedures.

After slaughter, birds were defeathered, eviscerated, and weighed to record the dressed carcass weight. Dressing percentage was calculated using the formula:

Dressing%=(Dressedcarcassweight/Livebodyweight)×100

The breast**,** thigh**,** liver**,** gizzard**,** heart**,** thymus**,** spleen**,** bursa of Fabricius**,** and abdominal fat were carefully excised and weighed using a digital balance with 0.01 g precision. Breast and thigh cuts were considered primary meat yield indicators, while the weights of liver, gizzard, and heart represented visceral organ development. Immune organs (thymus, spleen, and bursa) and abdominal fat were also recorded to assess health and fat deposition patterns. All carcass components were expressed in absolute weight (g). Abdominal fat was manually removed from the surrounding area of the cloaca and gizzard.

### Apparent nutrient digestibility

To assess the impact of dietary cinnamon and turmeric powder supplementation on nutrient digestibility, a digestibility trial was conducted during the final week of the experiment (day 36 to day 42). Four birds per treatment (one per replicate) were randomly selected and placed in individual metabolic cages specifically designed for fecal collection. These cages were equipped with wire mesh floors to facilitate separation of excreta from feed particles and to prevent contamination.

Before the collection period, a two-day adaptation phase was provided during which the selected birds were allowed to adjust to the metabolic cages and their respective diets. Following this, fecal samples were collected quantitatively for four consecutive days. During the collection period, feed and water were supplied ad libitum. The total feed intake was recorded for each bird, and fresh excreta were collected twice daily (morning and evening). The samples were pooled per bird, weighed, dried in a hot air oven at 60 °C for 72 hours, ground to a fine powder, and stored in airtight containers for chemical analysis. The proximate composition of both feed and fecal samples was analyzed following the standard procedures of the Association of Official Analytical Chemists (AOAC, 2005)**.** Parameters analyzed included dry matter (DM), crude protein (CP), crude fat (CF), and crude fiber (CFb).

$$\text{Apparent}\;\text{digestibility}=100-\left[100\;\times \frac{\text{Nutreint}\;\left(\%\right)\;\text{in}\;\text{digesta}\times \text{Cr}\left(\%\right)\text{in}\;\text{feed}}{\text{Nutrient}\left(\%\right)\text{in}\;\text{feed}\times \text{Cr}\left(\%\right)\text{in}\;\text{digesta}}\right]$$

Where Cr = chromic oxide

### Immune response assessment

Broiler chickens were vaccinated following a standard schedule for infectious bursal disease (IBD), infectious bronchitis (IB), and Newcastle disease (ND). To assess humoral immunity, 3 birds per replicate were randomly selected on day 42, and blood samples were collected from the wing vein. Serum was separated and stored at −20 °C. Antibody titers against IBD, IB, and ND were measured using commercial ELISA kits (IDEXX, USA). For SRBC titers, a 1 % SRBC suspension was injected intramuscularly on day 35, and titers were measured on day 42 using the hemagglutination assay, expressed as log₂ values.

### Renal function tests

At day 42 of the experimental period, blood samples were collected from the wing vein of broiler chickens (*n* = 6 per treatment group) using sterile syringes and allowed to clot at room temperature. Serum was separated by centrifugation at 3000 rpm for 10 minutes and stored at −20°C until analysis (Kalsoom et al., 2024). Renal function parameters including uric acid, urea, and creatinine concentrations were determined using commercially available diagnostic kits (Biolab, Pakistan) following the manufacturer’s instructions and analyzed spectrophotometrically using a UV–visible spectrophotometer (Model: UV-1800, Shimadzu, Japan).

### Statistical analysis

Prior to statistical analysis, all data were tested for normality using the Shapiro–Wilk test to ensure compliance with ANOVA assumptions. All data were statistically analyzed using one-way analysis of variance (ANOVA) under a completely randomized design (CRD) using SPSS software (Version 26.0; IBM Corp., Armonk, NY, USA). In this analysis, the dietary treatment was considered the fixed factor, while the replicate (pen) was treated as the experimental unit and considered the random (variable) factor. Treatment means were compared using Tukey’s post hoc test, and differences were considered statistically significant at *P* < 0.05. Results are presented as means ± standard error of the mean (SEM).

## Results

### Growth performance

The effect of dietary supplementation with cinnamon and turmeric powders on the growth performance of broiler chickens is presented in Table 3. Initial body weights were similar across all treatments at day 0 (*P* > 0.05). However, significant differences (*P* < 0.01) were observed in body weight gain (BWG), feed intake (FI), and feed conversion ratio (FCR) during all phases of the trial. From day 0 to 21, birds supplemented with 1.0 % cinnamon (T2) and 1.0 % turmeric (T4) showed significantly higher BWG and FI compared to the control. These groups also recorded the lowest FCR values, indicating better feed efficiency. A similar trend continued during the finisher phase (22–42 days), where treatments T2 and T4 again showed significantly superior BWG, FI, and FCR compared to other groups. Overall (0–42 days), birds in groups T2 and T4 demonstrated the highest cumulative BWG and FI, along with the lowest FCR values, followed by moderate improvements in groups T2 and T3, while the control group had the poorest performance. Mortality remained low and statistically non-significant across treatments.

**Table 3:** Effect of cinnamon and turmeric powders on growth performance of broiler chickens.

Performance Parameters	Treatments						
	Control	T1	T2	T3	T4	P-value	SEM
**0 – 21 Day**							
Initial body weight (g)	40.03 ± 0.166	40.02 ± 0.071	39.97 ± 0.144	40.06 ± 0.116	40.13 ± 0.154	0.540	0.067
Body weight gain (g)	902.50^c^ ± 5.465	961.33^b^ ± 4.724	1010.17^a^ ± 7.395	970.84^b^ ± 1.008	1021.15^a^ ± 14.909	<0.01	4.063
Feed intake (g)	1731.75^c^ ± 7.894	1750.45^b^ ± 10.872	1788^a^ ± 5.296	1757.21 *^a^* ± 2.875	1797.22^a^ ± 11.436	<0.01	4.169
FCR (g/g)	1.92^a^ ± 0.002	1.82^b^ ± 0.010	1.77^c^ ± 0.008	1.81^b^ ± 0.005	1.76^c^ ± 0.031	<0.01	0.008
**22 – 42 Day**							
Body weight gain (g)	1004.68^d^ ± 3.837	1132.32^b^ ± 3.942	1165.29^a^ ± 9.403	1094.56^c^ ± 1.955	1169.17^a^ ± 8.902	<0.01	3.176
Feed intake (g)	1892.09^d^ ± 7.081	2054.73^b^ ± 7.357	2084.34^a^ ± 6.407	1981.15^c^ ± 3.156	2090.40^a^ ± 9.305	<0.01	3.478
FCR (g/g)	1.88^a^ ± 0.008	1.81^b^ ± 0.004	1.79^c^ ± 0.011	1.81^b^ ± 0.004	1.79^c^ ± 0.006	<0.01	0.004
**0 – 42 Day**							
Body weight gain (g)	1907.18^d^ ± 26.838	2093.65^b^ ± 3.968	2175.46^a^ ± 4.188	2065.40^c^ ± 9.400	2190.32^a^ ± 8.087	< 0.01	6.735
Feed intake (g)	3623.84^d^ ± 83.146	3805.18^b^ ± 13.920	3872.34^a^ ± 36.125	3738.36^c^ ± 21.202	3887.62^a^ ± 23.772	< 0.01	21.710
FCR (g/g)	1.90^a^ ± 0.0258	1.82^b^ ± 0.0096	1.78^c^ ± 0.0141	1.81^b^ ± 0.0082	1.77^c^ ± 0.0126	< 0.01	0.008
Mortality %	2.5 ± 5.00	0.00 ± 0.000	0.00 ± 0.000	2.5 ± 5.00	0.00 ± 0.000	0.573	1.581

Means in same row labelled with different superscripts are different significantly at *p* < 0.05; Control (fed basal diet without supplements); T1: 0.5 % Cinnamon powder; T2: 1.0 % cinnamon powder; T3: 0.5 % turmeric powder; T4: 1.0 % turmeric powder; SEM: Standard Error of Mean.

### Carcass traits

The effect of dietary cinnamon and turmeric powder supplementation on carcass traits of broiler chickens at day 42 is presented in Table 4. Significant improvements (*P* < 0.01) were observed in dressed carcass weight, dressing percentage, breast and thigh meat yields, and abdominal fat content across the treatments. Broilers fed diets supplemented with 1.0 % cinnamon (T2) and 1.0 % turmeric (T4) exhibited the highest dressed carcass weights and dressing percentages, significantly outperforming the control group. Similarly, breast and thigh weights were markedly increased in these groups, with group T4 yielding the highest breast meat and T2 the highest thigh mass, indicating enhanced muscle deposition.

**Table 4:** Effect of cinnamon and turmeric powders on carcass traits of broiler chickens at day 42.

Carss traits parameters weight (g)	Treatments						
	Control	T1	T2	T3	T4	*P*-value	SEM
Dressed carcass	1328.4^d^ ± 6.04	1484.5^c^ ± 6.57	1593.3^a^ ± 5.05	1475.1^b^ ± 4.44	1600.1^a^ ± 4.58	< 0.01	2.700
Dressing %	67.48^e^ ± 0.311	69.30^d^ ± 0.307	71.60^b^ ± 0.227	69.70^c^ ± 0.211	72.00^a^ ± 0.206	< 0.01	0.128
Breast	337.28^d^ ± 2.451	393.01^c^ ± 2.894	410.30^a^ ± 1.432	399.67^b^ ± 2.733	410.92^a^ ± 1.275	< 0.01	1.130
Thigh	150.80^d^ ± 1.005	190.07^b^ ± 0.952	206.38^a^ ± 1.566	187.58^c^ ± 1.870	204.86^a^ ± 1.174	< 0.01	0.680
Liver	42.45 ± 1.289	41.71 ± 0.791	41.80 ± 1.220	42.64 ± 1.445	41.22 ± 1.081	0.461	0.593
Gizzard	44.70 ± 0.635	44.30 ± 0.946	44.11 ± 1.051	43.91 ± 1.002	43.98± 0.942	0.763	0.463
Heart	11.73 ± 0.539	11.29 ± 0.553	11.16 ± 0.635	11.57 ± 0.618	11.57 ± 0.380	0.611	0.276
Thymus	2.63 ± 0.196	2.58 ± 0.273	2.67 ± 0.145	2.56 ± 0.133	2.58 ± 0.187	0.931	0.097
Spleen	2.62 ± 0.064	2.63 ± 0.069	2.70 ± 0.055	2.64 ± 0.024	2.67 ± 0.041	0.280	0.027
Bursa	3.62 ± 0.017	3.63 ± 0.013	3.63 ± 0.017	3.62 ± 0.037	3.63 ± 0.047	0.951	0.015
Abdominal fat	40.50^c^ ± 0.511	30.75^b^ ± 0.660	26.53^a^ ± 0.679	31.01^b^ ± 0.895	26.10^a^ ± 0.562	< 0.01	0.337

Means in same row labelled with different superscripts are different significantly at *p* < 0.05; Control (fed basal diet without supplements); T1: 0.5 % Cinnamon powder; T2: 1.0 % cinnamon powder; T3: 0.5 % turmeric powder; T4: 1.0 % turmeric powder; SEM: Standard Error of Mean.

On the other hand, internal organ weights (liver, gizzard, heart, thymus, spleen, and bursa) were not significantly influenced (*P* > 0.05) by any dietary treatment. However, abdominal fat was significantly reduced in birds receiving 1.0 % cinnamon (T2) and 1.0 % turmeric (T4), showing the lowest values compared to the control, suggesting improved lipid metabolism or fat deposition control.

### Apparent nutrients digestibility

The effect of dietary supplementation with cinnamon and turmeric powders on apparent nutrient digestibility in broiler chickens is shown in Table 5. A significant improvement (*P* < 0.01) was observed in the digestibility of dry matter, crude protein, crude fat, and crude fiber among the supplemented groups compared to the control. The highest digestibility values for all measured nutrients were recorded in birds fed 1.0 % cinnamon (T2) and 1.0 % turmeric (T4).

**Table 5:** *E*ffect of cinnamon and turmeric powders on apparent nutrient digestibility of broiler chickens.

Nutrient digestibility parameters (%)	Treatments						
	Control	T1	T2	T3	T4	P-value	SEM
Dry Matter	67.59^d^ ± 0.718	72.23^b^ ± 1.113	74.71^a^ ± 0.506	70.52^c^ ± 0.890	74.29^a^ ± 0.686	< 0.01	0.405
Crude Protein	53.40^c^ ± 0.433	56.38^b^ ± 0.446	58.56^a^ ± 0.449	55.83^b^ ± 0.311	58.44^a^ ± 0.439	< 0.01	0.209
Crude Fat	66.54^d^ ± 0.449	68.52^c^ ± 0.862	71.56^a^ ± 0.978	70.16^b^ ± 0.593	72.02^a^ ± 1.134	< 0.01	0.421
Crude Fibre	8.05^d^ ± 0.026	9.85^c^ ± 0.119	11.90^a^ ± 0.091	9.82^c^ ± 0.103	11.68^b^ ± 0.025	< 0.01	0.042

Means in same row labelled with different superscripts are different significantly at *p* < 0.05; Control (fed basal diet without supplements); T1: 0.5 % Cinnamon powder; T2: 1.0 % cinnamon powder; T3: 0.5 % turmeric powder; T4: 1.0 % turmeric powder; SEM: Standard Error of Mean.

### Immune response

The immunomodulatory effects of cinnamon and turmeric powder supplementation on antibody titers in broiler chickens are presented in Table 6**.** Birds receiving either spice supplementation, particularly at 1.0 % inclusion levels (T2 and T4), exhibited significantly (*P* < 0.01) elevated antibody titers against IBD, IB, ND, and SRBC, compared to the control group. For IBD titers, the highest values were observed in group T2 and T4, whereas the control group showed the lowest response. Similar patterns were observed for IB and ND titers, with group T4 (1.0 % turmeric) and T2 (1.0 % cinnamon) producing significantly greater responses than lower-dose or control groups. In SRBC titers (Log₂), the highest immune response was recorded in T2 and T4, followed by a moderate increase in groups T1 and T3, compared to the lowest response in the control group.

**Table 6:** Antibodies titers against infectious bursal disease, infectious bronchitis, Newcastle disease and sheep red blood cells of broiler chickens fed cinnamon and turmeric powders.

Antibodies titers	Treatments						
	Control	T1	T2	T3	T4	P-value	SEM
Infectious Bursal Disease	2512.30^e^ ± 2.513	2720.90^c^ ± 0.401	3424.75^a^ ± 1.349	2715.66^d^ ± 1.425	3422.29^b^ ± 1.298	< 0.01	0.775
Infectious Bronchitis	62.93^e^ ± 1.074	67.14^d^ ± 0.525	70.91^b^ ± 0.5996	68.52^c^ ± 0.492	72.17^a^ ± 0.552	< 0.01	0.342
Newcastle Disease	40.83^e^ ± 0.496	45.90^c^ ± 0.219	50.72^b^ ± 0.562	44.60^d^ ± 0.216	51.60^a^ ± 0.698	< 0.01	0.239
Sheep Red Blood Cell titers Log_2_	5.23^d^ ± 0.031	6.81^c^ ± 0.013	9.09^a^ ± 0.017	6.79^c^ ± 0.022	9.05^b^ ± 0.022	< 0.01	0.011

Means in same row labelled with different superscripts are different significantly at *p* < 0.05; Control (fed basal diet without supplements); T1: 0.5 % Cinnamon powder; T2: 1.0 % cinnamon powder; T3: 0.5 % turmeric powder; T4: 1.0 % turmeric powder; SEM: Standard Error of Mean.

### Renal function tests

The effects of cinnamon and turmeric powder supplementation on renal function parameters in broiler chickens at day 42 are summarized in Table 7. A significant (*P* < 0.01) reduction in serum uric acid levels was observed in all treatment groups (T1 to T4) compared to the control. Birds fed the basal diet without supplementation had the highest uric acid concentration, whereas the lowest values were recorded in T2 and T4, both supplemented with 1.0 % cinnamon and turmeric, respectively.

**Table 7:** Effect of cinnamon and turmeric powders on serum uric acid, urea and creatinine of broiler chickens at day 42.

Renal function parameters	Treatments						
	A	B	C	D	E	P-value	SEM.
uric acid (mg/dl)	12.42^a^ ± 0.079	7.01^b^ ± 0.024	6.96^b^ ± 0.017	7.00^b^ ± 0.057	6.95^b^ ± 0.021	< 0.01	0.023
Urea (mg/dl)	5.08 ± 0.141	5.04 ± 0.127	5.00 ± 0.083	4.98 ± 0.108	5.02 ± 0.141	0.824	0.061
Creatinine (mg/dl)	0.162 ± 0.015	0.160 ± 0.022	0.160 ± 0.018	0.160 ± 0.008	0.152 ± 0.013	0.917	0.008

Means in same row labelled with different superscripts are different significantly at *p* < 0.05; Control (fed basal diet without supplements); T1: 0.5 % Cinnamon powder; T2: 1.0 % cinnamon powder; T3: 0.5 % turmeric powder; T4: 1.0 % turmeric powder; SEM: Standard Error of Mean.

## Discussion

The observed improvements in BWG, FI, and FCR in broilers supplemented with 1.0 % cinnamon and 1.0 % turmeric powders align with previous findings on the growth-promoting properties of these phytogenic additives. The enhanced performance is likely due to the bioactive compounds—cinnamaldehyde and eugenol in cinnamon, and curcumin in turmeric—which exert antimicrobial, antioxidant, and digestive-stimulant effects (Tajodini et al., 2015; Ali et al., 2021; Bilal et al., 2022).These compounds improve gut health by modulating microflora, stimulating digestive enzymes, and enhancing nutrient digestibility. Improved FI in cinnamon-fed birds may reflect better palatability, while enhanced FCR corresponds with improved nutrient absorption (Naderi et al., 2014). Curcumin also supports intestinal morphology and bile acid secretion, aiding in nutrient absorption and lipid metabolism (Rajput et al., 2013; Li et al., 2023). The effects were more pronounced at 1.0 % inclusion, likely because higher concentrations deliver more bioactive compounds, surpassing the threshold needed to activate physiological pathways and yield measurable benefits. In contrast, lower doses may provide insufficient concentrations for full biological impact.

Carcass trait improvements, including higher dressing percentage and muscle yields and reduced abdominal fat, were significant at 1.0 % inclusion. These effects reflect enhanced protein synthesis and lipid metabolism regulated by compounds such as cinnamaldehyde and curcumin through pathways like AMPK and PPAR-γ (Shehzad et al., 2011; Khaafi et al., 2024). Cinnamon reduces lipogenesis and enhances lipid oxidation, while turmeric promotes lean tissue accretion and reduces fat deposition (Hussein, 2013; Wang et al., 2015). At lower doses, these regulatory effects may be less robust due to suboptimal activation of metabolic targets.

Improved nutrient digestibility in groups receiving 1.0 % cinnamon and turmeric may be due to enhanced secretion of pepsin, trypsin, bile, and lipase, promoting protein and fat utilization (Khan et al., 2012; El-Hamid et al., 2024). Higher doses likely exert stronger effects on enzyme activity and microbial balance, increasing fiber degradation and nutrient uptake (Tajodini et al., 2015; El-Gogary et al., 2025).

The immunomodulatory effects of cinnamon and turmeric were evident from elevated antibody titers against IBD, ND, and SRBC at 1.0 % inclusion. Their polyphenolic content enhances cytokine production, lymphocyte activation, and MHC expression via NF-κB and MAPK pathways (Rajput et al., 2013; Ali et al., 2021). These responses were dose-dependent, as higher concentrations provide more potent stimulation of immune functions (Qasem et al., 2015; Khanghahi et al., 2024).

A significant reduction in serum uric acid in the high-dose groups suggests better renal efficiency and lower oxidative stress. Curcumin and cinnamaldehyde protect renal tissues by reducing inflammation and ROS production, thus improving uric acid clearance (Cai et al., 2022; Abdeen et al., 2019). The absence of changes in urea and creatinine implies normal kidney function across all groups, while the specific reduction in uric acid may reflect improved purine metabolism (Elazab et al., 2021; Abubakar et al., 2023).

## Conclusion

Dietary supplementation with 1.0 % cinnamon or turmeric powder significantly improved growth performance, feed efficiency, carcass yield, nutrient digestibility, immune function, and renal health in broiler chickens. These enhancements are attributed to the bioactive compounds in cinnamon and turmeric that promote gut health, enhance enzyme activity, and modulate immune and metabolic responses. From a practical perspective, these findings support the inclusion of cinnamon and turmeric as effective natural alternatives to synthetic growth promoters, especially in light of increasing restrictions on antibiotic use in poultry production. Their use can contribute to sustainable and health-conscious broiler farming by enhancing productivity without compromising animal welfare or product safety.

## Disclosure statement

No potential conflict of interest was reported by author(s)

## Ethical approval

The Committee on Animal Rights and Welfare, The University of Agriculture Peshawar, Pakistan approved this study (FAHVS/122/2023).

## Data availability

Data will be made available from the authors upon reasonable request.

## CRediT authorship contribution statement

**Khanzada Khan:** Data curation. **Hanan Al-Khalaifah:** Resources. **Nazir Ahmad:** Conceptualization, Project administration, Supervision. **Muhammad Tahir Khan:** Investigation, Software. **Rasha Alonaizan:** . **Rifat Ullah Khan:** Validation, Visualization, Writing – original draft, Writing – review & editing. **Shabana Naz:** Methodology. **Ala Abudabos:** Formal analysis. **Ibrahim A. Alhidary:** Funding acquisition.

## Disclosures

Authors declare no conflict of interest
